# Localization of ABCG5 and ABCG8 proteins in human liver, gall bladder and intestine

**DOI:** 10.1186/1471-230X-4-21

**Published:** 2004-09-21

**Authors:** Eric L Klett, Mi-Hye Lee, David B Adams, Kenneth D Chavin, Shailendra B Patel

**Affiliations:** 1 Division of Endocrinology, Diabetes and Medical Genetics, Medical University of South Carolina, STR 541, 114 Doughty Street, Charleston, South Carolina 29403, USA; 2 Division of Gastrointestinal Surgery, Medical University of South Carolina, 96 Jonathan Lucas Street, CSB 211, Charleston, SC 29425, USA; 3 Division of Transplant Surgery, Medical University of South Carolina, 96 Jonathan Lucas Street, CSB 404, Charleston, SC 29425, USA

## Abstract

**Background:**

The molecular mechanisms that regulate the entry of dietary sterols into the body and their removal via hepatobiliary secretion are now beginning to be defined. These processes are specifically disrupted in the rare autosomal recessive disease, Sitosterolemia (MIM 210250). Mutations in either, but not both, of two genes *ABCG5 *or *ABCG8*, comprising the *STSL *locus, are now known to cause this disease and their protein products are proposed to function as heterodimers. Under normal circumstances cholesterol, but not non-cholesterol sterols, is preferentially absorbed from the diet. Additionally, any small amounts of non-cholesterol sterols that are absorbed are rapidly taken up by the liver and preferentially excreted into bile. Based upon the defects in sitosterolemia, ABCG5 and ABCG8 serve specifically to exclude non-cholesterol sterol entry at the intestinal level and are involved in sterol excretion at the hepatobiliary level.

**Methods:**

Here we report the biochemical and immuno-localization of ABCG5 and ABCG8 in human liver, gallbladder and intestine using cell fractionation and immunohistochemical analyses.

**Results:**

We raised peptide antibodies against ABCG5 and ABCG8 proteins. Using human liver samples, cell fractionation studies showed both proteins are found in membrane fractions, but they did not co-localize with caveolin-rafts, ER, Golgi or mitochondrial markers. Although their distribution in the sub-fractions was similar, they were not completely contiguous. Immunohistochemical analyses showed that while both proteins were readily detectable in the liver, ABCG5 was found predominately lining canalicular membranes, whereas ABCG8 was found in association with bile duct epithelia. At the cellular level, ABCG5 appeared to be apically expressed, whereas ABCG8 had a more diffuse expression pattern. Both ABCG5 and ABCG8 appeared to localize apically as shown by co-localization with MRP2. The distribution patterns of ABCG5 and ABCG8 in the gallbladder were very similar to each other. In the small intestine both ABCG5 and ABCG8 appear to line the brush border. However, at the level of the enterocyte, the cellular distribution patterns of ABCG5 and ABCG8 differed, such that ABCG5 was more diffuse, but ABCG8 was principally apical. Using standard deglycosylation methods, ABCG5 and ABCG8 do not appear to be glycosylated, suggesting a difference between human and mouse proteins.

**Conclusion:**

We report the distribution patterns of ABCG5 and ABCG8 in human tissues. Cell fractionation studies showed that both proteins co-fractionated in general, but could also be found independent of each other. As predicted, they are expressed apically in both intestine and liver, although their intracellular expression patterns are not completely congruent. These studies support the concept of heterodimerization of ABCG5 and ABCG8, but also support the notion that these proteins may have an independent function.

## Background

The gastrointestinal tract is the initial barrier to dietary constituents and is important in regulating nutrient entry, as well as keeping non-nutrients out. Additionally, the hepatobiliary system acts as an additional filter to rapidly excrete such non-nutrients into bile, thus keeping the net retention of these potential toxins low. Mammals have evolved many mechanisms in the gastrointestinal tract to select out usable dietary constituents from those that maybe potentially toxic to the body. It is apparent that the ATP-binding cassette proteins (ABC proteins/transporters) are the machinery that mediate the ATP-dependent transport of a wide variety of substrates that range from xenobiotics to peptide fragments [1]. A subset of these ABC transporters, located in the canalicular membranes of mammalian liver, play key roles in bile formation and detoxification [1-3].

One of these processes involves the regulation of sterol entry and excretion. Whole body cholesterol homeostasis is a tightly regulated process, involving dietary absorption, *de novo *synthesis and hepatobiliary secretion. Sitosterolemia, a rare autosomal recessive disorder of sterol metabolism results in the disruption of dietary sterol entry and hepatobiliary sterol secretion [4-6]. Under normal circumstances, our diets contain equal amounts of plant sterols and cholesterol, but the plant sterols are specifically excluded from our bodies and only regulated amounts of cholesterol are retained. In Sitosterolemia, this exclusion is defective resulting in the retention of non-cholesterol sterols. Mutations in either, but not both, of two ABC transporters, ABCG5 and ABCG8, encoded by a single locus, *STSL*, are known to cause this disease [7-9]. Based upon the genetics, as well as *in vitro *and *in vivo *data, these 'half-transporters' are proposed to function as obligate heterodimers. *In vitro *experiments have shown that both proteins are needed to be co-expressed for apical expression and that these may function as mutual chaperones in the ER for maturation [10,11]. *In vivo *experiments in mice have not been consistent. Using the Abcg5/Abcg8 double knockout mice, Graf et al has shown that by inoculating them with adenoviral constructs for Abcg5 and Abcg8 that both are required for expression of both proteins [11]. Additionally, Plosch *et al *and our group have constructed mouse models deficient in either Abcg5 [12] or Abcg8 [13] that show different biliary physiology than that of the Abcg5/Abcg8 double knockout mice. This suggests these proteins may have independent function(s) in addition to their function as heterodimers. However, to date, no reports of characterization and localization of the human proteins have been reported.

In this report, we examined the location of these two proteins using cellular fraction and immunohistochemical analyses of human liver, gallbladder and small intestine. We found a general concordance of co-expression of both proteins, but we also noted that ABCG5 and ABCG8 could be found in plasma membranes, as well as in intracellular membrane locations independent of each other. Additionally, deglycosylation of human liver membranes with peptidyl N-glycosidases did not alter the mobility of the proteins after SDS-PAGE, suggesting that these proteins may not be glycosylated in human liver. This differential localization suggests that perhaps ABCG5 and ABCG8 may have functions independent of each other, as well as functioning as heterodimers.

## Methods

Tissue aquistion. Human liver donated for transplantation, but deemed unsuitable for transplantation on inspection by the transplant service (usually based upon a 'fatty' appearance) was obtained in accordance with IRB approval. As soon as the liver was deemed unsuitable (typically less than 10 h following harvesting) pieces were either snap-frozen in liquid nitrogen and stored at -80°C or liquid nitrogen until use, or placed in ice-cold 2-methylbutane and stored in liquid nitrogen. Samples from more than nine different donors were used in these studies. Additionally, human gallbladders and segments of proximal small intestine were obtained from patients under going either laproscopic cholecystectomy or pancreatoduodenectomy (Whipple procedure). These tissues were directly taken from the operating room in normal saline on ice to be processed directly for frozen sectioning.

### Antibodies

Anti-membrin, anti-transferrin, and anti-calnexin antibodies were obtained from Stressgen (Victoria, BC Canada), anti-caveolin antibody from BD Bioscience (San Diego, CA, USA), anti-MDR1 that also detects MDR2/3 (C219) from Centocor Inc. (Malvern, PA, USA), anti-MRP2 (cMOAT) from Chemicon International (Temecula, CA, USA) and secondary antibodies were purchased from Jackson Immuno research (West Grove, PA, USA). Polyclonal rabbit anti-sera to human ABCG5 and ABCG8 peptides were generated in-house, using a 20-peptide immunogen from human ABCG5 (576–587, FQKYCSEILVVNEFYGNFTC, GenBank Accession number NP_071881) and a 22-peptide sequence from human ABCG8 (608–629, SRRTYKMPLGNLTIAVSGDKIL, GenBank Accession number NP_071882). The anti-sera were further purified using peptide affinity columns and stored at a concentration of 0.8 mg/ml in Immuno Pure Binding Buffer (Pierce, Rockford, IL, USA). For peptide blocking experiments the peptides were dissolved in DMSO (final concentration 30%), incubated with the corresponding peptide for 1 1/2 hours at 37°C then used for immunoblotting as described below.

### Membrane protein preparation

Crude total membrane isolation was carried out with minor modifications as previously described [14]. All the procedures were carried out at 4°C. Three grams of human liver were homogenized in homogenization buffer (5 mM Tris pH7.5, 250 mM sucrose, 1 mM PMSF, 20 μg/μl of leupeptin and 1 μg/μl of aprotinin) by applying 10 strokes with a dounce homogenizer. The homogenate was centrifuged at 1000 *g *for 10 minutes, the pellet containing any undisrupted cells and nuclear debris were re-homogenized with one-half the initial volume of homogenization buffer, centrifuged at 1000 *g *for 10 minutes and this process was repeated once more. Supernatants were pooled and subjected to centrifugation at 100,000 *g *for 40 minutes. The resulting pellet, deemed the crude membrane fraction, was used as the starting material for Western blotting and fractionation experiments.

### Nycodenz gradient fractionation

Human liver crude total membrane proteins were re-suspended in 30% Nycodenz solution (Nycodenz in 5 mM Tris-HCl pH 7.5 and 1 mM EDTA). This suspension was loaded on top of a 40% Nycodenz solution cushion in an ultracentrifuge tube, overlaid by consecutive 23%, 20%, 15% and 10% Nycodenz solutions and subjected to centrifugation at 39,000 rpm for 16 hours at 4°C in a SW41 rotor (Beckman Instrument, Palo Alto, CA). After centrifugation, 800–1000 μl fractions were sequentially removed from the top, combined with two volumes of homogenization buffer (see above) and centrifuged at 39,000 rpm at 4°C for 40 minutes to remove the Nycodenz. The resulting pellets were re-suspended in buffer (25 mM Tris-HCl pH 7.5, 150 mM NaCl, 0.1% Triton X-100 and 0.1% SDS, 1 mM PMSF, 20 μg/μl of leupeptin and 1 μg/μl of aprotinin), the protein content determined by the method of Lowry and fractions analysed by SDS-PAGE. Equal amounts of protein (25 μg) per lane were loaded.

### Sucrose gradient fractionation

The procedure for membrane fractionation was essentially as described for the Nycodenz fractionation, except for the homogenization buffer used (25 mM Tris pH6.8, 150 mM NaCl, 1 mM PMSF, 20 μg/μl of leupeptin and 1 μg/μl of aprotinin). The sucrose density gradient fractionation was modified as previously described [15-17]. Human liver crude membrane proteins were re-suspended in 1% Triton X100 buffer (25 mM Tris-HCl pH6.5, 150 mM NaCl, 1% Triton X100, 1 mM PMSF, 20 μg/μl of leupeptin and 1 μg/μl of aprotinin), adjusted to a final sucrose concentration of 40% and overlaid with a 15–30% linear sucrose gradient. The samples were subjected to centrifugation at 39,000 rpm for 16 hours at 4°C in a SW41 rotor (Beckman Instrument, Palo Alto, CA, USA) and fractions collected from the top as described above. The proteins from fractions 1–4 from top of the tube were precipitated with acetone because these fractions did not contain sufficient protein for direct analysis. After protein concentrations were determined, equal amounts of proteins (20 μg) from each fraction were resolved by SDS-PAGE.

### Immunoblotting

Proteins resolved by SDS-PAGE were transferred onto nitrocellulose membranes. Membranes were then blocked for 1 hour in 5% dry milk in PBS-T (Phosphate Buffered saline and 0.1% Tween 20) and incubated with primary antibody against either ABCG5 or ABCG8 in 5% milk in PBS-T overnight at 4°C. Blots were washed three times for 5 minutes in TBS-T (Tris Buffered Saline/0.1% Tween-20) with 150 mM NaCl, incubated with goat-anti-rabbit conjugated HRP antibodies (1:10000 dilution), washed for three times 5 minutes and developed with Western Lightning^® ^Chemiluminescence Reagent Plus (Perkin Elmer Life Sciences, Inc. Boston, MA, USA).

### Immunohistochemical analysis and microscopy

Snap-frozen liver, gallbladder and intestine tissues were used to cut 8 μm thick frozen sections, air-dried for 30 minutes onto glass slides and kept at -80°C until used. The slides were stained with hematoxylin, rinsed with PBS three times, fixed for 10 minutes with cooled methanol at -20°C and rinsed with PBS three times. The slides were treated with blocking solution (10% donkey serum in 0.1 M glycine/PBS) for 30 minutes at room temperature and incubated with primary antibody overnight at 4°C. The slides were washed with PBS and incubated with secondary antibody (goat-anti-rabbit conjugated with Cy3™ or rhodamine or FITC) for 20–30 minutes at room temperature, rinsed with PBS three times and examined under an Olympus BX-5 confocal microscope with Fluoview.

## Results

### Identification of ABCG5 and ABCG8 in crude total membrane preparations of human liver

**Figure 1:**
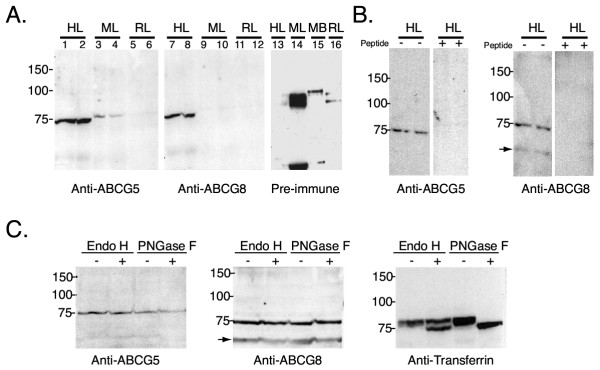
**Immunoblotting analyses of ABCG5 and ABCG8 in human liver. **Panel A shows the immunodetection of ABCG5 (tracks 1–6), ABCG8 (tracks 7–12) in membrane preparations from human liver (HL, tracks 1, 2, 7, 8) mouse liver (ML, tracks 3, 4, 9, 10) or rat liver (RL, tracks 5, 6, 11, 12). The anti-ABCG5 peptide antibody detected a faint mouse band, but no other specific binding was identified. Although the pre-immune sera detected bands in the rodent tissue samples, none were detected in human liver (tracks 13–16, MB, mouse brain). Specificity was further shown by pre-incubation of the antibodies with the peptides they were raised against (panel B). In the presence of the specific peptides, the 75 kDa bands are not detected in human liver microsomes. Panel C shows the results of deglycosylation of human total liver microsomes, probed with anti-ABCG5 (left hand panel), anti-ABCG8 (middle panel) or anti-transferrin (right hand panel). Aliquots from the same incubation were separated for all three western blots. Although ABCG5 and ABCG8 do not appear to have their SDS-PAGE mobility's altered by either EndoH or PNGase F treatment, that of transferrin in the same samples is clearly effected (see Text for discussion). Newly synthesized (sensitivity to EndoH), as well as mature forms of transferrin (resistant to EndoH, but fully sensitive to PNGase F) are present in the liver membrane preparations.

Peptide antibodies were raised against human ABCG5 and human ABCG8 and affinity purified prior to use (see Methods). The immunogen peptides used for the antibodies were selected since they were sequences that lay outside of the predicted transmembrane domains and based upon antigenicity.Western blotting experiments (Figure 1A) showed that both anti-ABCG5 and anti-ABCG8 antibodies bound to ~75 kDa proteins in human liver crude membranes. Pre-immune sera did not detect the ~75 kDa expected bands. Pre-incubation of the immune antibodies with the peptides against which they were raised abolished specific binding (Figure 1B). For anti-ABCG5 5 μg of peptide was needed to block 1 μg of antibody and for anti-ABCG8 12.5 μg of peptide was needed to block 1 μg of antibody. Interestingly, a ~60 kDa band was detected using the anti-ABCG8 antibody whose signal is abolished when incubated with the peptide from which the antibody was raised (Figure 1B and 1C, arrow indicated band). The significance of this is unclear at present. These antibodies were also tested against mouse and rat liver membrane preparations and no significant cross-reactivity was detected except for faint bands seen with anti-ABCG5 in mouse liver samples (Figure 1A, tracks 3 and 4). No other bands were detected above the 150 kDa marker in all western blots. Interestingly, while these proteins are predicted to be N-glycosylated [8,9], only single bands in the appropriate molecular weight range were detected and no higher molecular bands were observed. To investigate whether these proteins are glycosylated, crude membrane fractions were digested with EndoH, PNGase F and examined for alterations in gel migration by SDS-PAGE (Figure 1C). Although the mobility of a known glycoprotein, transferrin, was increased in the same fractions following deglycosylation, there was no change in the mobility of ABCG5 or ABCG8 (Figure 1C).

### Localization of ABCG5 and ABCG8 by Nycodenz and Sucrose gradient fractionation of human liver

**Figure 2:**
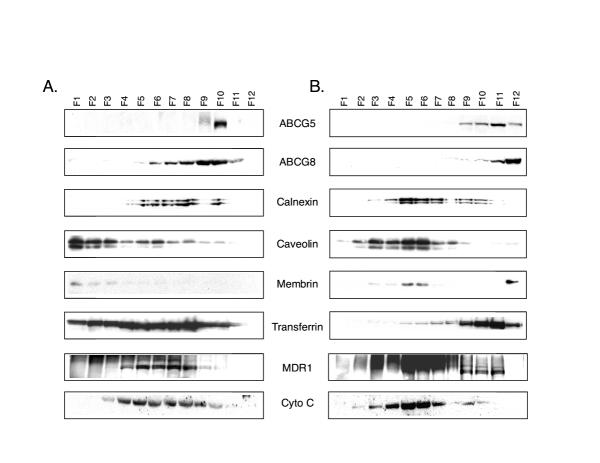
**Subcellular localization of ABCG5 and ABCG8 in human liver. **Panel A shows the Nycodenz gradient fractionation and panel B the Triton X-100/sucrose gradient fractionation. A representative result from each of these is shown. F1–F12 represents serial fractions collected from the top of the tube. Proteins from each of these fractions were separated by SGS-PAGE, western blotted and probed for the proteins as indicated on the figure. Calnexin (ER marker), membrin (Golgi marker) and caveolin (raft marker) did not co-localize with ABCG5/ABCG8 when both biochemical fractionation patterns are compared. However, MDR1 (apical membrane marker) and transferrin (plasma membrane marker) showed some consistency with ABCG5/ABCG8 co-localizations (tracks F9–10, panel A and F11, panel B).

Crude total membrane proteins from human liver were fractionated by Nycodenz gradient centrifugation and examined for localization markers by western blot analyses (Figure 2A). After Nycodenz gradient centrifugation, ABCG5 (fractions 9–10) and ABCG8 (fractions 6–11) were found to have a broad range of distribution and appeared to be distributed in a pattern similar to calnexin (an ER membrane marker, fractions 5–10), Cytochrome C (a mitochondrial marker, fractions 4–9), transferrin (a plasma membrane marker, fractions 1–11), caveolin (fractions 6–10) and MDR1 (an apical membrane marker, fractions 4–10). ABCG5 and ABCG8 did not co-localize with cis-Golgi (Figure 2A) markers.

To examine whether ABCG5 and ABCG8 were associated with membrane rafts, total membrane proteins from human liver were solubilized with ice-cold 1% Triton X-100 detergent and fractionated by sucrose density gradient centrifugation (Figure 2B). Fractionation resulted in two Triton X-100 insoluble complexes, as judged by the clarity of the gradient fractions. The first was found in the low-density range (15–20% sucrose, fractions 2–6, Figure 2B) and the second in the high-density range (40% sucrose, fractions 10–12, Figure 2B). Caveolin-rich fractions localized to the low-density range (Figure 2B, fractions 2–6). However, ABCG5 and ABCG8 were detected in the high-density fractions, F10–12, along with transferrin (fractions 5–12) and MDR1 (fractions 9–11). Calnexin or cytochrome C, under these conditions did not co-localize with either ABCG5 or ABCG8. Note that the majority of the ABCG5 and ABCG8 were present in the densest fractions, F11–12. Under these conditions, membrin, a cis-Golgi membrane marker, was also detected in fraction 12.

Thus, ABCG5 and ABCG8 have significant overlap with each other suggesting co-localization. However, these proteins did not seem to co-localize with any specific membrane marker except transferrin, when two different methods of fractionation were utilized.

### Immunohistochemical localization of ABCG5 and ABCG8 in human liver

**Figure 3:**
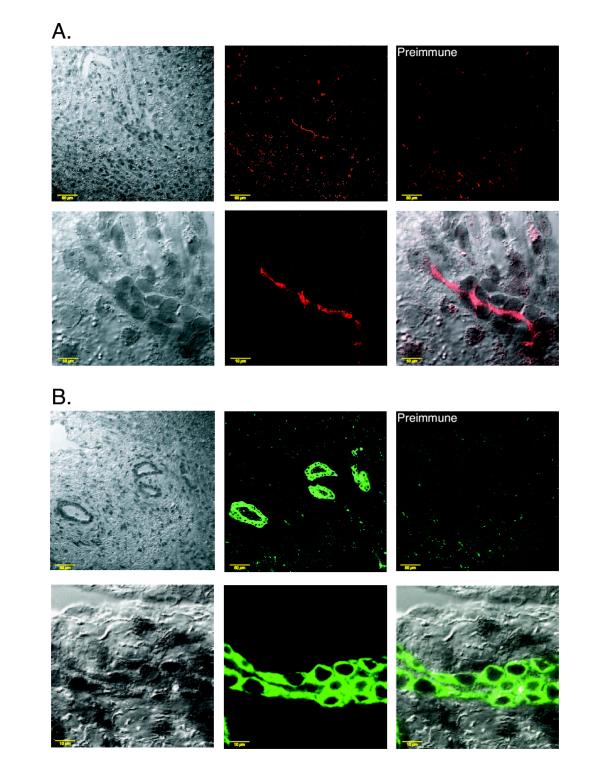
**Immunolocalization of ABCG5 and ABCG8 in human liver sections.** Panel A shows the staining pattern of ABCG5 and panel B that for ABCG8. The pre-immune controls for both antibodies are as marked and shown in the top right hand corners of each panel. The top panels of each section are at low magnification (bar is 50 μm) and the bottom panels at high magnification (10 μm). The images for ABCG5 and ABCG8 were visualised with red and green colors respectively using Adobe Photoshop (Adobe, Cupertino, CA). The left panels show hematoxylin stained phase contrast images and the middle panels show the fluorescence images after immune serum staining. The bottom right panel of each section shows the merged images of phase contrast and the fluorescence signals. ABCG5 was readily detectable in canalicular cells and at higher magnification seemed to be apical in expression (panel A). On the other hand, ABCG8 was more readily detectable in cells lining the bile ducts (panel B, top panels), as well as in canalicular cells; although its cellular expression appeared more diffuse (see Text for discussion).

**Figure 4:**
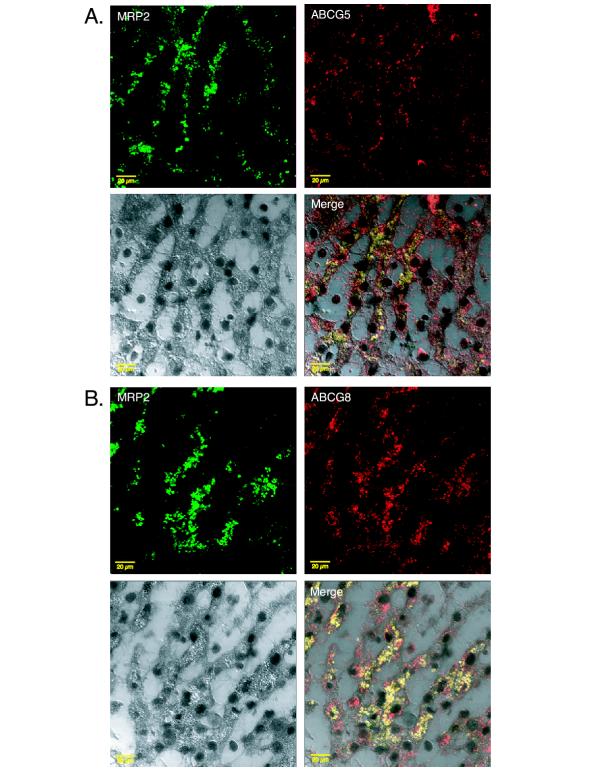
**Co-localization of ABCG5 and ABCG8 with MRP2 by immunohistochemistry. **Liver sections were simultaneously incubated with either the combination of anti-ABCG5/MRP2 or anti-ABCG8/MRP2. Panel A shows staining patterns of MRP2 and ABCG5 independently (upper portion) then merged together (lower right) with what appears to be similar over-lapping expression patterns. Likewise MRP2 and ABCG8 expression patterns appear to over lap as well as shown in panel B. Bar is 20 μm.

It has been shown previously that ABCG5 and ABCG8 are expressed only in the liver and intestine [8,9]. To further characterize the location of ABCG5 and ABCG8 in human liver, immunohistochemical analyses were performed on frozen serial sections of human liver. Pre-immune sera were used as negative controls. The distribution of the two proteins appeared to be divergent not only histologically, but also at the cellular level. From a histological point of view, ABCG5 was detected along sinusoidal tracts (Figure 3A, upper panel) whereas ABCG8 was detected within the cells lining the bile ducts (Figure 3B, upper panel). At higher magnification, ABCG5 was detected along bile canaliculi and at the cellular level appeared to be an apically expressed (Figure 3A, lower panel). However, the distribution of ABCG8 at the cellular level appeared more diffuse consistent with plasma membrane expression and perhaps in intracellular membranes (Figure 3B, lower panel). Expression of ABCG5 within intracellular vesicular compartments could not be excluded by the techniques employed. To confirm an apical location of ABCG5 and ABCG8 immunohistochemical co-localization studies were carried out using an antibody against a known apical transporter (MRP2) in the liver. As shown in Figure 4, ABCG5 and ABCG8 have significant overlap in expression with MRP2 (panels A and B, respectively).

### Immunohistochemical localization of ABCG5 and ABCG8 in human gall bladder

**Figure 5:**
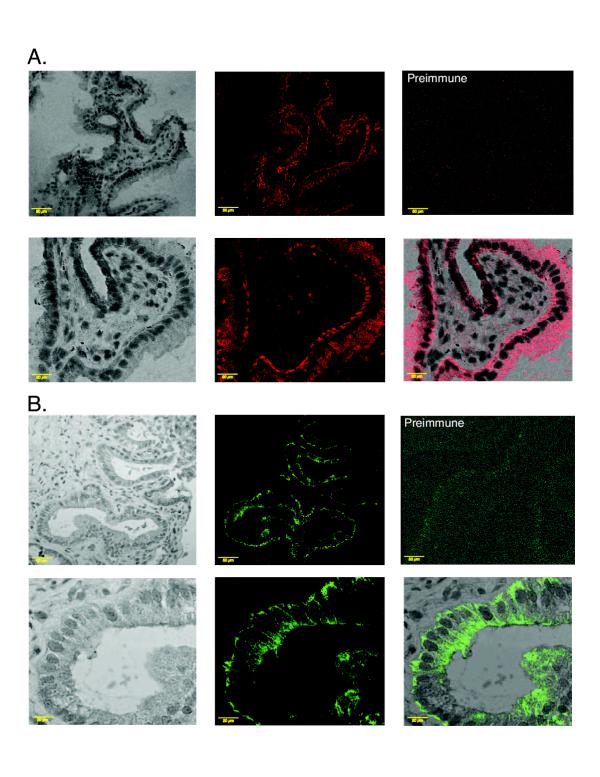
**Immunolocalization of ABCG5 and ABCG8 in human gall bladder sections. **Gall bladder surgical samples were stained for ABCG5 (panel A, red staining) or ABCG8 (panel B, green staining). The layout is as indicated for figure 3. Both ABCG5 and ABCG8 appeared to be confined to the epithelial cells lining the lumen and no significant staining in any of the deeper cell layers was detected. At the cellular level, both proteins seem to be diffusely expressed within the cell and at plasma membrane. A strict apical expression was not observed for either of these proteins.

Serial sections of human gall bladder were incubated with each antibody and pre-immune serum was used as a negative control. Both ABCG5 and ABCG8 were detected in the epithelium of gall bladder mucosa (Figure 5A and 5B). At higher magnification, the cellular distribution of the signals detected was similar and showed a diffuse cytoplasmic distribution (Figure 5A and 5B).

### Immunohistochemical localization of ABCG5 and ABCG8 in human small intestine

**Figure 6:**
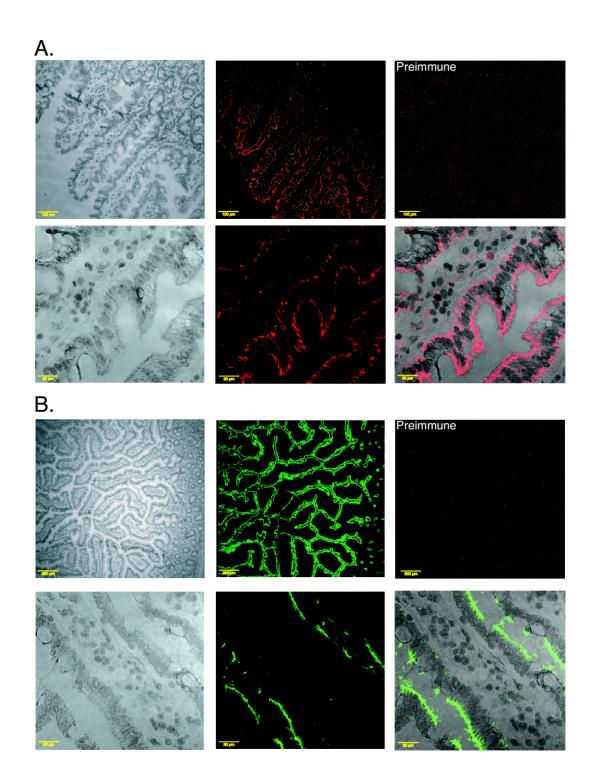
**Immunolocalization of ABCG5 and ABCG8 in human intestinal sections. **Human intestinal surgical samples were stained with ABCG5 (panel A, red staining) or ABCG8 (panel B, green staining). The layout is as indicated for figure 3. At the cellular level, ABCG5 was expressed in a more diffuse pattern (panel A, see bottom right hand panel). In contrast, ABCG8 was expressed in the apical surfaces of the enterocytes (panel B, bottom right hand panel). Both proteins seemed to be expressed only in the enterocytes lining the villi and no significant expression was detected in any of the other cell layers.

Both ABCG5 and ABCG8 were detected in the apical surfaces of the enterocytes in biopsy samples of the small intestine (Figure 6A and 6B, upper panels). However, at higher magnification the cellular distribution of ABCG5 appeared more diffuse (Figure 6A, lower panel) whereas ABCG8 was expressed apically (Figure 6B, lower panel). This divergent pattern was seen in all of the serial sections analyzed.

## Discussion

In this study, we report the localization of ABCG5 and ABCG8 in human liver, gall bladder and intestine. Our studies showed that these proteins are highly specific in the cells they are expressed. In the liver, expression is seen in cells lining the hepatobiliary tracts, both hepatocytes and ductal cells. In the intestine, robust expression was seen only in the villus enterocyte layers. In the gall bladder, expression was confined to the epithelial cells lining the lumen. However, some differences in the distribution of ABCG5 and ABCG8 within these tissues were apparent. In the liver, ABCG8 was highly expressed in the hepatocytes lining the bile ducts, whereas ABCG5 was more robustly expressed in hepatocytes lining the cannaliculae. In fractionation studies, using two different methods of separation, the distribution of ABCG5 and ABCG8 was compatible with both proteins potentially acting as heterodimers. However, we also noted that there were fractions where only one of these proteins, but not the other was detected. This could be an artefact, with one antibody being a better reagent, or that this pattern could truly reflect that each of these proteins can also exist independently, perhaps as homodimers. Overall, the distribution patterns of ABCG5 and ABCG8 in these cellular fractionations were similar to that observed with the plasma membrane marker transferrin and apical membrane marker MDR1. Additionally, immunohistochemical analyses show that ABCG5 and ABCG8 are apically expressed in the liver, gall bladder and intestine. In the liver ABCG5 and ABCG8 also appear to co-localize with the known apical protein MRP2. This confirms previous data from Graf *et al*, using *in vitro *expression in WIF-B cells [10], and support the contention that ABCG5 and ABCG8 are plasma membrane proteins.

These data would suggest that expression of these proteins might not be wholly dependent upon mutual co-expression, as has been reported for the mouse and in *in vitro *studies [10,11]. However, one note of caution should be expressed. All of the liver samples analyzed were obtained because they were unsuitable for transplantation. Most livers were considered to be 'fatty' livers. While these livers were not effectively diseased, that fact that they had fatty infiltrates may have influenced the normal expression of these two half-transports. Thus, confirmation in normal human liver samples will be needed, though this may not be feasible.

With that reservation in mind, our data do have important implications for sterol trafficking in humans.

Firstly, the relatively robust and highly specific expression of ABCG5 and ABCG8 in gall bladder epithelium confirms the important role of this organ in regulating biliary secretion. In addition to the production of bile by the liver, the gall bladder may be able to further regulate the sterol content of bile, via ABCG5/ABCG8 activity. A similar pattern of expression has been reported in canine gall bladder epithelial cell culture and these data confirm these findings in human gall bladder [18].

Secondly, the differences in expression patterns of ABCG5 and ABCG8 in liver, gall bladder and intestine, though subtle, seem to indicate the several important possibilities. It is not clear which organ is of paramount importance in the human in regulating non-cholesterol sterol retention. And it is not clear if ABCG5 and ABCG8 play a significant role in determining cholesterol entry at the intestinal level, though they seem to be implicated strongly as determining sterol excretion at the level of the hepato-biliary system. In mice deficient for Abcg5/Abcg8 or Abcg5, cholesterol absorption rates were not dramatically affected [12,19,20]. Hepatobiliary excretion of all sterols was significantly (but not completely) reduced. In mice singly deficient for Abcg5 or Abcg8, some differences have been reported [12,13]. In Abcg5 KO mice, following LXR activation, sterol excretion in bile was comparable to wild-type mice, though it is not clear if this also restored plant sterol excretion. In contrast, although cholesterol absorption studies for Abcg8 KO mouse have not been reported, biliary excretion of cholesterol was dramatically reduced and no stimulation of excretion was observed after forced bile acid infusions. While all of these data have been reported using different protocols (LXR activation, bile acid infusions, or static gall bladder puncture), these data suggest that there exist other pathways for sterol trafficking in both liver and intestine. At present it would be speculative to assume that these 'other' pathways involve ABCG5 or ABCG8 as homodimers, but this possibility is supported by the circumstantial evidence of separate patterns of expression of human ABCG5 and ABCG8 in these tissues.

Finally, although rodent sterolins are glycosylated and *in vitro *glycosylation is readily demonstrable, human ABCG5 and ABCG8 did not appear to be glycosylated as judged by deglycosylation-migration assays. It is possible that this technique is insensitive and these proteins are glycosylated. Alternatively, it is possible that the antibodies we have raised only react with unglycosylated forms and thus fail to detect the glycosylated forms. With respect to the first issue, deglycosylation-migration has been demonstrated to detect mouse glycosylated proteins and since these proteins are highly conserved, this possibility seems remote. With respect to the second possibility, if our antibodies were exclusively detecting unglycosylated (and presumably immature forms), the apical patterns of expression of these proteins in both the liver and intestine would seem to suggest that these proteins seem to traffic to these specialized membranes normally. In the absence of an independent method, and the lack of a direct assay for function, whether these proteins form an active heterodimer can not be resolved at present.

## Conclusion

In summary, we report the first immunolocalization of ABCG5 and ABCG8 in human liver, gall bladder and intestine. Our data show that these proteins are located in membranes and can have an apical expression in all of these tissues. Biochemical, as well as immunolocalization studies show that while both proteins co-localize in general, they can also seem to have expression patterns that may be independent of each other.

## Competing interests

None declared.

## Authors' contributions

ELK and MHL performed the experiments, KDC and DBA provided the liver and surgical samples respectively SBP was responsible for supervision, data analyses and obtaining funding for these experiments. ELK and SBP wrote the paper.

## Pre-publication history

The pre-publication history for this paper can be accessed here:


